# Time and productivity loss associated with immunotherapy infusions for the treatment of melanoma in the United States: a survey of health care professionals and patients

**DOI:** 10.1186/s12913-022-08904-4

**Published:** 2023-02-09

**Authors:** Raquel Aguiar-Ibáñez, Emilie Scherrer, Dmitri Grebennik, John Cook, Shalini Bagga, Baanie Sawhney, Anvi Khandelwal, Scott A. Soefje

**Affiliations:** 1 Merck Canada Inc., Kirkland, QC Canada; 2 Merck & Co., Inc., Rahway, NJ USA; 3 Complete Health Economics and Outcomes Research Solutions, North Wales, PA USA; 4 Mayo Clinic, Rochester, MN USA

**Keywords:** Immunotherapies, Pembrolizumab, Nivolumab, Survey, Productivity loss, Infusion visit

## Abstract

**Introduction:**

A new dosing schedule for the oncology immunotherapy pembrolizumab, every 6 weeks (Q6W), has been approved by the U.S. FDA, reducing the frequency of visits to infusion centers. We quantified the time spent by oncologists, nurses, patients, and caregivers per melanoma-related immunotherapy infusion visit to evaluate its potential impact.

**Methods:**

Surveys were self-completed by 100 oncologists, 101 oncology nurses, and 100 patients with melanoma across the U.S. to quantify the time spent per infusion visit with pembrolizumab (Q3W or Q6W), nivolumab (Q2W or Q4W), or nivolumab+ipilimumab (nivolumab in combination: Q3W; nivolumab maintenance: Q2W or Q4W). Time measures included traveling, waiting, consultation, infusion, post-treatment observation, and caregiving. Respondents were also surveyed regarding the impact of the COVID-19 pandemic on infusion treatments.

**Results:**

Responses deemed valid were provided by 89 oncologists, 93 nurses, and 100 patients. For each new [returning] patient treated with pembrolizumab, nivolumab or nivolumab+ipilimumab, oncologists reported to spend an average of 90 [64], 87 [60] and 101 [69] minutes per infusion visit (*p*-value for between-group difference = 0.300 [0.627]). For first [subsequent] treatment cycles, nurses reported spending 160 [145] average minutes per visit for nivolumab+ipilimumab, versus roughly 120 [110] for the single agents (*p*-value for between-group difference = 0.018 [0.022]). Patients reported to spend an average of 263, 382, and 224 minutes per visit at the center for pembrolizumab (*N* = 47), nivolumab (*n* = 34), and nivolumab+ipilimumab (*n* = 15) respectively (*p*-value for between-group difference = 0.0002). Patients also reported that their unpaid (*N* = 20) and paid caregivers (*N* = 41) spent with them an average of 966 and 333 minutes, respectively, from the day before to the day after the infusion visit.

**Conclusion:**

Less frequent immunotherapy infusion visits may result in substantial time savings for oncologists, nurses, patients, and caregivers.

**Supplementary Information:**

The online version contains supplementary material available at 10.1186/s12913-022-08904-4.

## Introduction

There is a need to optimize the efficiency of oncology-related infusions at cancer centers to improve patient flow, decrease time patients spend to receive treatment, and increase patients’ satisfaction [1, 2]. Approaches have been developed and tested showing the potential impact of achieving these goals [3–5], and some centers have adopted the Oncology Care Model (OCM) aiming to improve the infusion delivery process. The OCM is structured as an episode-based payment model that combines fee for service, monthly payments for additional care, and ties payments to performance based on meeting certain quality metrics and practice transformation requirements. The model aims to improve the quality and coordination of care for cancer patients at a lower cost [6].

In the United States (U.S.) pembrolizumab and nivolumab are immunotherapies approved by the Food and Drug Administration (FDA) for the treatment of patients with unresectable or metastatic melanoma (nivolumab as a single agent or in combination with ipilimumab) [7, 8]. As single agents, pembrolizumab and nivolumab are also approved as an adjuvant treatment for patients with melanoma with involvement of lymph node(s) following complete resection [7, 9]. The approved dosing regimen for nivolumab monotherapy is 240 mg Q2W or 480 mg Q4W [7]. When combined with ipilimumab, the approved dosing for nivolumab is 1 mg/kg when initially combined with ipilimumab 3 mg/kg Q3W for 4 doses, and then 240 mg Q2W or 480 mg Q4W [7, 8].

In 2020, the FDA approved a new dosing schedule for pembrolizumab which reduces the frequency of administration from 200 mg Q3W to 400 mg Q6W in all existing adult indications [9, 10], including cutaneous melanoma, the most deadly type of skin cancer [11, 12]. Less frequent dosing can reduce the use of healthcare resources, lessen the administrative burden on infusion centers, and increase patient satisfaction [2, 3, 5]. Characterizing and quantifying the burden of infusion visits from three key perspectives - oncologists, oncology nurses, and patients- can help medical decision making. While there are published estimates of patients’ wait time and planned time for chemotherapy infusions [1, 2], data is lacking for a comprehensive assessment of the burden of infusion visits for immunotherapies. To the best of our knowledge, neither the time spent by oncologists and oncology nurses (nurses henceforth) during an immunotherapy infusion event, nor the overall time spent by patients or the associated time losses borne by them and their caregivers have been examined.

We conducted nationwide U.S. surveys to better understand the burden associated with immunotherapy infusions. The purpose was to assess the time oncologists and nurses spend during each phase of a typical infusion visit with new and returning patients [1], as well as the travel time, wait time, chair time, and post-observation time spent by patients and their caregivers per infusion visit. The study also estimated the loss of productivity of patients and their caregivers by capturing time lost in terms of paid work and unpaid household work, in addition to lost leisure time. Our study focused on the most prescribed immunotherapies for advanced melanoma in the U.S., i.e., pembrolizumab, nivolumab, and nivolumab+ipilimumab. MEK and BRAF inhibitors were not included as these are oral treatments that specifically target patients with BRAF mutation positive tumors. The surveys (provided as Data Supplement) also explored the perceptions of patients, physicians, and nurses regarding the impact of the COVID-19 pandemic on the infusion visit.

## Methods

### Study design and participants

Distinct surveys were developed for oncologists, nurses and patients to inquire about the time spent during an immunotherapy infusion visit when treating melanoma (in the adjuvant or the metastatic settings) with either pembrolizumab, nivolumab, or nivolumab+ipilimumab (the latter only for unresectable or metastatic disease) [7, 9]. A pilot test of each questionnaire was performed on 21 oncologists, 14 nurses, and 16 patients to gather feedback on the comprehension of the questions. Afterwards, the questionnaires were finalized by retaining or improving the language that allowed better comprehension. Respondents were then recruited by Schlesinger Quantitative across the U.S. via online channels, and the surveys were conducted [13]. Participants were recruited from double-opted verified panelists (the respondent first agreed to the panel and privacy rules to opt into the panel; Schlesinger then used an email confirmation and government-issued ID to double opt-in and verify). Panelists received a personalized email invitation for this study that included general survey details such as survey reference number, length of the online survey, device compatibility, and incentive. The first set of questions in each online survey were used to determine whether the participants fulfilled the inclusion criteria described below. If a respondent did not meet the inclusion criteria, the survey was terminated, and the respondent was excluded from the study.

Oncologists and nurses prescribing/administering at least one of the above-mentioned treatments for melanoma, and patients 18 years-old and above, with stage III or IV melanoma, were eligible for voluntary participation. Patients diagnosed with any other cancer were excluded. Between December 2020 and February 2021 oncologists, nurses and patients self-completed the questionnaires via an online platform [13]. Recruitment quotas of 100 patients, 100 oncologists, and 100 nurses were pre-specified. The sample of patients was further targeted to represent 50 patients receiving adjuvant therapy after having undergone complete resection (30 on pembrolizumab and 20 on nivolumab), and 50 treated in the metastatic setting (20, 15, and 15 patients treated with pembrolizumab, nivolumab and nivolumab+ipilimumab, respectively). Nurses were asked to report time by dose-specific immunotherapy regimen since they support patients across the full infusion event and were anticipated to be able to report more granular details about the time spent at different stages in the infusion process. Given that oncologist are not involved in each of the activities related to an infusion event, they were asked to report time estimates per patient and infusion visit by immunotherapy type, as it was anticipated they would be able to report less granular details.

Patients reported the time spent during the infusion visit including travel time. Patients were also asked about the time lost from paid work, unpaid household work, and leisure time for a typical infusion visit. Patients answered these questions for themselves and also their perception of the time spent by their paid and unpaid caregivers on the day before, the day of, and the day after the infusion visit. Productivity loss was measured in terms of both work loss and household chore time lost (by the patient and/or their caregiver).

### Statistical analysis

Kruskal–Wallis tests were performed to assess significant differences in time estimates across immunotherapies (pembrolizumab, nivolumab, and nivolumab+ipilimumab, regardless of dosing schedule) and within dosing schedules per immunotherapy (the latter for time measures reported by nurses for pembrolizumab Q3W versus Q6W, and for nivolumab Q2W versus Q4W). A significant between-group difference indicated that the times for at least one of the immunotherapies or schedules would differ from the others.

### Compliance with ethics guidelines

No administration of any therapeutic or prophylactic agent or medical procedures was required as part of this study. The protocol was exempted from the Institutional Review Board (IRB) as determined by Advarra [14].

## Results

### Study samples

In total, 100 oncologists, 101 nurses and 100 patients completed the surveys. Questionnaires from nurses and oncologists who provided the same answer across all the questions or that reported unrealistic long times (as assessed by SS) for drawing blood, checking vitals, infusion, or consultation were deemed invalid. In addition, individual responses to question by patients were excluded if deemed invalid (e.g., time lost due to an infusion visit exceeded 24 hours within a day, unrealistic responses such as total time at the center being shorter than infusion times, non-reconciliation of travel time with the distance between home and infusion center). Of the 89 oncologist surveys deemed valid, 81 (91.0%), 82 (92.1%), and 85 (95.5%) oncologists provided answers for pembrolizumab, nivolumab, and nivolumab+ipilimumab treatments, respectively. Of the 93 nurse surveys deemed valid, 86 (92.5%), 85 (91.4%), and 83 (89.2%) provided answers for pembrolizumab, nivolumab, and nivolumab+ipilimumab treatments, respectively. None of the patient surveys were entirely excluded, although some responses were considered invalid and excluded from the analysis.

### Health care professional characteristics

Oncologists worked most often in private practice (41 [46.1%]), followed by academic medical centers (25 [28.1%]), community hospitals (21 [23.6%]), and private hospitals (2 [2.2%]). Among nurses, 50 (53.8%) were from academic medical centers, 21 (22.6%) from private practice, and 18 (19.4%) from community hospitals. The centers at which the surveyed health care professionals practiced were mainly in the Northeast (18 oncologists [20.2%] and 41 nurses [44.1%]) and Midwest (36 oncologists [40.4%] and 19 nurses [20.4%]), with the remaining centers located in the South and the West (35 oncologists [39.3%] and 33 nurses [35.5%]) [15]. A total of 32 oncologists [36.0%] and 39 nurses [41.9%] reported practicing under the OCM. The number of infusion chairs at the centers ranged between 3 and 125 (average of 23.8) for oncologists and between 5 and 100 (average of 31.4) for nurses. Nurses reported seeing on average between 4 and 99 patients each day (mean: 50.4).

### Patient characteristics

Patients who responded had a mean age of 41.8 years-old, 87.0% were males, and all were employed. Fewer patients were from the Northeast (10 [10.0%]) versus the South (38 [38.0%]), West (27 [27.0%]), and Midwest (38 [38.0%]) [15]. In total, 25 patients (out of 100) reported receiving treatments in an academic setting, 71 in a non-academic setting, and 4 did not know. Among patients treated with pembrolizumab, 10 (20.0%) received the Q6W regimen, and among those treated with nivolumab, 5 (14.3%) received the Q4W regimen.

### Time spent per infusion by health care professionals and patients

**Fig. 1:**
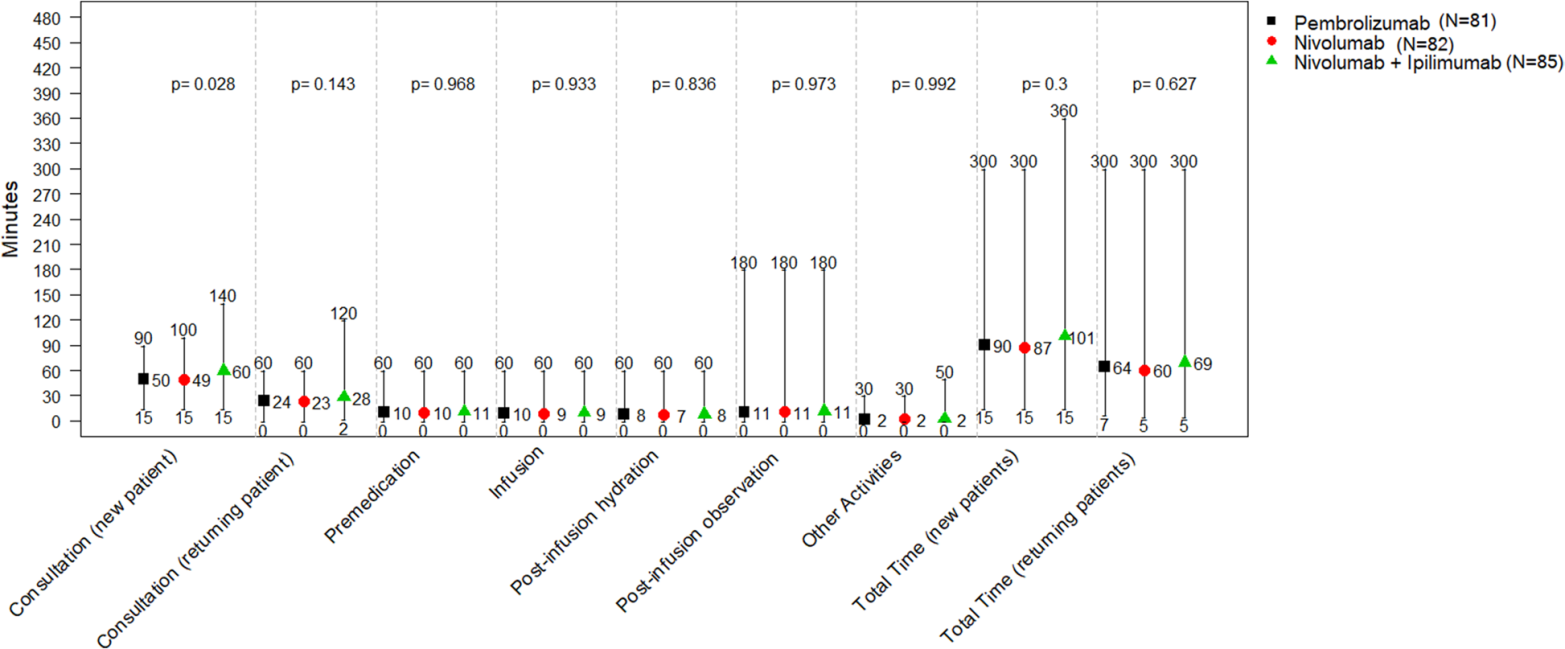
Mean, minimum, and maximum time (in minutes) oncologists spend per patient and infusion visit by immunotherapy regimen administered. *P*-values assess if differences by immunotherapy regimen are significant

Figure 1 shows the time oncologists reported to spend per infusion visit for new and returning patients. On average, oncologists reported to spend 90, 87 and 101 minutes per infusion for new patients treated with pembrolizumab, nivolumab, and nivolumab+ipilimumab, respectively (*p*-value for between-group difference: 0.300), and just over an hour for returning patients (64, 60 and 69 minutes for pembrolizumab, nivolumab, and nivolumab+ipilimumab patients; *p*-value for between-group difference: 0.627).

**Fig. 2:**
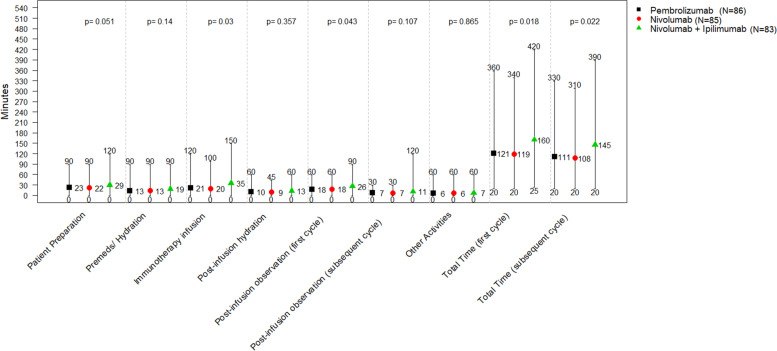
Mean, minimum, and maximum time oncology nurses spend per patient and infusion visit by immunotherapy administered. P-values assess if differences by immunotherapy are significant

Figure 2 shows the time nurses reported to spend per patient for each phase of an infusion visit. The mean post-observation time for first and subsequent cycles was 26 and 11 minutes with nivolumab+ipilimumab, and 18 and 7 minutes, respectively, with both pembrolizumab and nivolumab. The mean total time that nurses reported to spend per patient treated with pembrolizumab, nivolumab and nivolumab+ipilimumab was 121, 119, and 160 minutes for a first treatment cycle, and 111, 108 and 145 minutes for a subsequent cycle, respectively (*p*-value for between-group difference: 0.018 and 0.022, respectively). Specifically, the mean times nurses reported to spend for the immunotherapy infusion visit per patient receiving pembrolizumab, nivolumab and nivolumab+ipilimumab were 21, 20 and 35 minutes, and 18, 18 and 26 minutes for post-infusion observation for patients receiving a first treatment cycle (*p*-values for between-group difference: 0.030 and 0.043, respectively). When the two dosing schedules within a given immunotherapy were compared, no significant differences in time were observed, demonstrated by the similar point estimates and non-significant *p*-values. For pembrolizumab Q3W versus Q6W, the mean total infusion time was 121 versus 122 minutes (all p-values were > = 0.877) and for nivolumab Q2W versus Q4W, the mean total infusion time was 119 versus 120 minutes (all p-values were > = 0.863). Similar point estimates were observed by dosing schedule for the individual infusion activities with no significant differences (Fig. S1 in Supplementary File 2).

**Fig. 3:**
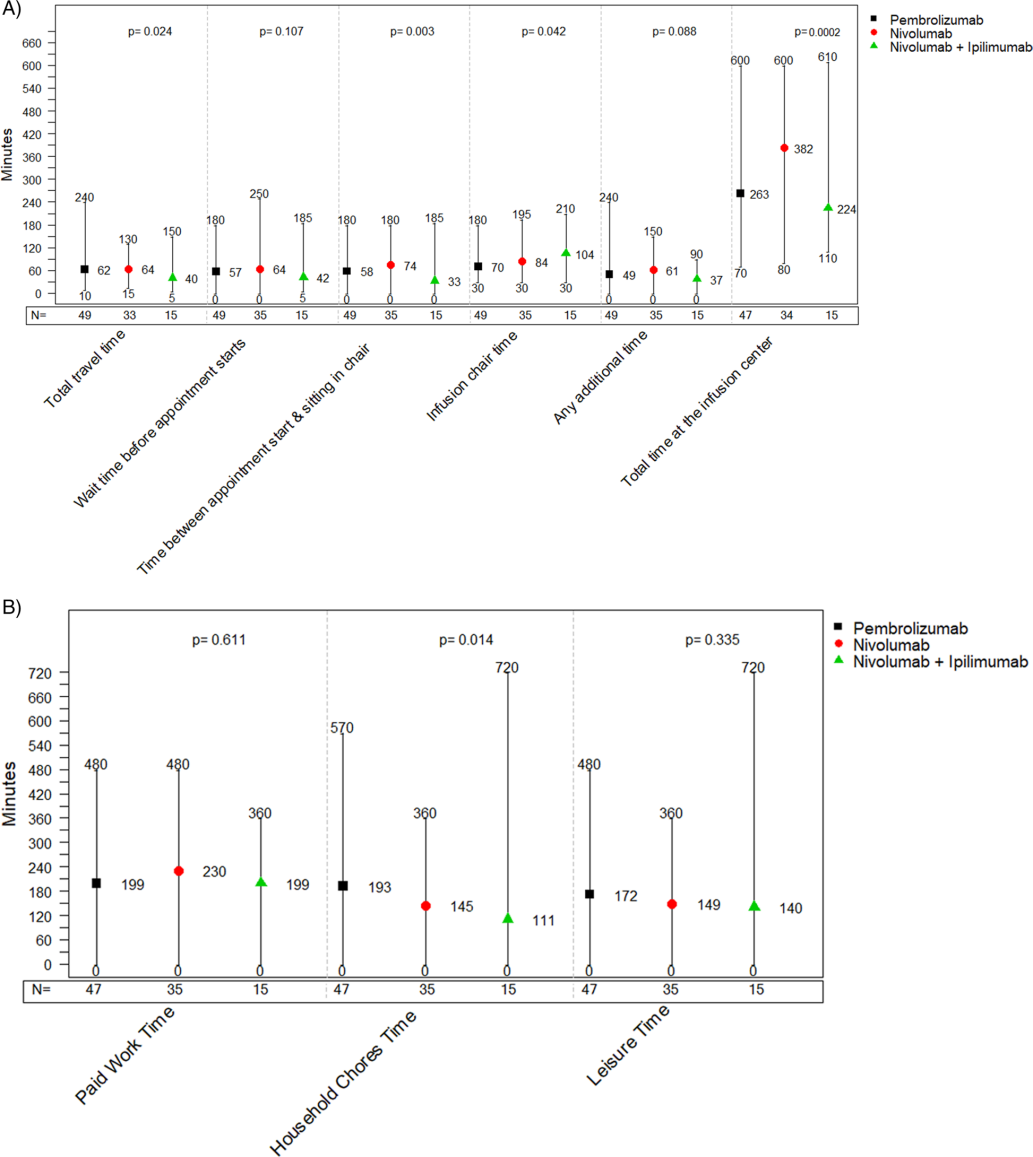
Average, minimum, and maximum time patients with melanoma spend per infusion visit by immunotherapy administered (**A**) by phase of the infusion visit and (**B**) by type of time loss on the day of infusion

Figure 3a illustrates the differences in time patients treated with each immunotherapy reported to spend travelling to and from the center, and at the center for each infusion-related activity. The mean reported time for travelling was 62, 64, and 40 minutes for patients treated with pembrolizumab, nivolumab and nivolumab+ipilimumab, respectively (*p*-value for between-group difference: 0.024). The mean time patients treated with pembrolizumab, nivolumab, and nivolumab+ipilimumab reported to wait between their arrival at the center and until they were seen by a physician or a nurse was 57, 64, and 42 minutes, respectively (*p*-value for between-group difference: 0.107). The mean infusion chair time reported per patients treated with pembrolizumab, nivolumab and nivolumab+ipilimumab was 70, 84 and 104 minutes, respectively; *p*-value for between-group difference: 0.042). In total, the mean time (in minutes) patients reported to spend at the center per infusion visit was 263 for pembrolizumab, 382 for nivolumab, and 224 for nivolumab+ipilimumab (p-value for between-group difference: 0.0002). The mean infusion chair time with pembrolizumab Q3W, pembrolizumab Q6W, nivolumab Q2W, nivolumab Q4W, and nivolumab+ipilimumab was 70, 56, 88, 58, and 104 minutes, respectively. In total, the mean time (in minutes) spent by patients at the center per infusion visit were 284 for pembrolizumab Q3W, 184 for pembrolizumab Q6W, 380 for nivolumab Q2W, 400 for nivolumab Q4W, and 224 for nivolumab+ipilimumab (Fig. S2 in Supplementary File 2).

As shown in Fig. 3b, patients treated with pembrolizumab, nivolumab and nivolumab+ipilimumab reported they missed on the day of infusion an average of 199, 230, and 199 minutes for paid work, 193, 145, and 111 minutes for household chores, and 172, 149, and 140 minutes for leisure, respectively (*p*-value for between-group difference: 0.611, 0.014, and 0.335, respectively).

### Caregiver time and overnight accommodation

**Fig. 4:**
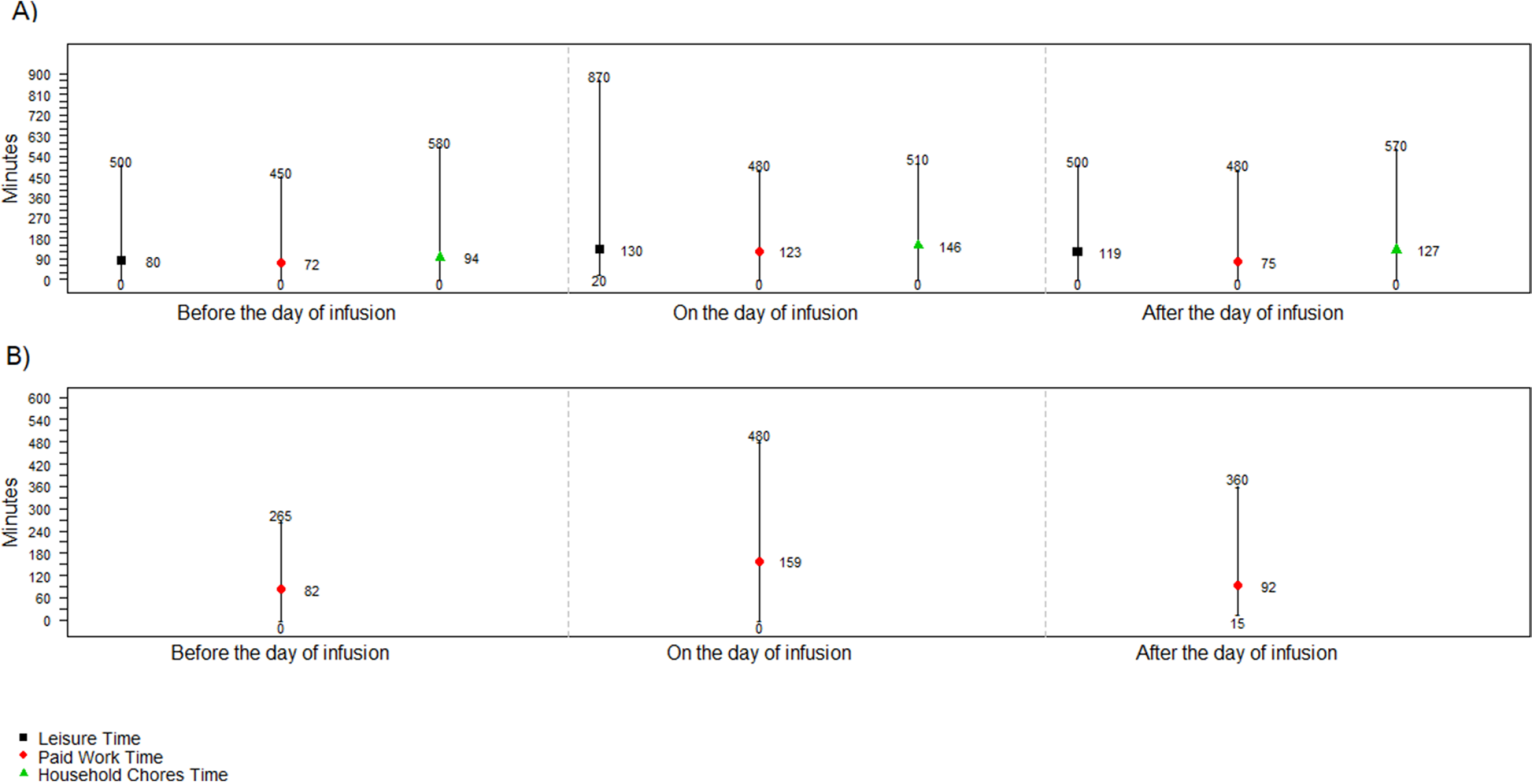
Average, minimum, and maximum time per infusion visit that patients reported their caregivers spent with them: for (**A**) Unpaid caregivers (*N* = 20) and (**B**) Paid caregivers (*N* = 41)

Twenty patients (20.0%) reported having an unpaid caregiver who missed an average of 270 minutes of paid work, 367 minutes of unpaid work/household chores, and 329 minutes of leisure time, from the day before through the day after the infusion (Fig. 4). A total of 41 patients (41.0%) reported having a paid caregiver who spent on average 159, 82, and 92 minutes per infusion visit on the day of infusion, the day before, and the day after, respectively (Fig. 4). Approximately half of the patients either always (28.0%) or sometimes (19.0%) required overnight accommodation.

### Impact of the COVID-19 pandemic

The majority of health care professionals (48 oncologists [53.9%] and 87 nurses [93.5%]) reported that COVID-19 influenced the typical infusion visit, with the three most commonly reported impacts being: changes in the time required for the infusion process (25 oncologists [52.1%] and 50 nurses [57.5%]), the availability of infusion time slots (20 oncologists [41.7%] and 35 nurses [40.2%], respectively) and changes in the number of patients undergoing infusions (with 16 oncologists [33.3%] and 30 nurses [34.5%] reporting changes in the number of new patients, and 17 oncologists [35.4%] and 22 nurses [25.3%] for returning patients). Other reported impacts of the COVID-19 pandemic included constraints with respect to visitors or caregivers accompanying the patient for their infusions (6 oncologists [12.5%] and 24 [27.6%] nurses) and more time required to maintain COVID-19 protocols (5 oncologists [10.4%] and 11 nurses [12.6%]). While most patients reported their infusion experience was not impacted by the pandemic, 14 (14.0%) reported a change in waiting time, 11 (11.0%) reported having difficulties booking appointments, 9 (9.0%) reported a change in the mode of transportation and travel time, and 4 (4.0%) reported an impact on the availability of caregivers.

## Discussion

The recent approval by the U.S. FDA of a new PD1 checkpoint inhibitor dosing schedule, pembrolizumab Q6W, can help health care administrators improve the operating efficiency of cancer centers. To quantify the potential benefits of a less frequent immunotherapy dosing schedule, we documented the time spent during an infusion visit through nationwide surveys in the U.S., involving oncologists and oncology nurses treating melanoma with pembrolizumab, nivolumab or nivolumab+ipilimumab, as well as patients with melanoma treated with these immunotherapies. To our knowledge, our study is the first to estimate time associated with infusion visits using the newer immunotherapies.

Our findings indicate that each infusion visit requires a substantial amount of time from oncologists (approximately 1.5 hours for new patients and 1 hour for returning patients), nurses (approximately 2 hours on average) and patients (on average, approximately 1 hour for travel and 5 hours at the center). Both oncologists and nurses report to spent longer times for new patients compared to returning patients (30 additional consultation minutes by oncologists and 10 extra minutes in post observation by nurses, per patient receiving their first treatment cycle). The time patients reported to spend per single agent immunotherapy infusion was aligned with what would be expected in the U.S., considering the recommended 30 minutes infusion for these treatments [7, 9] and other intra-venous infusions typically lasting 35 to 46 minutes according to the NCCN Infusion Efficiency Workgroup [1].

Nurses reported less time spent for patients treated with a monotherapy versus those treated with nivolumab+ipilimumab, especially with respect to the immunotherapy infusion. While the median infusion time reported by patients for the combination of 120 minutes was in line with recommendations (30 minute infusion for nivolumab followed by a 90 minute infusion period for ipilimumab) [7, 8], the mean infusion time was 104 minutes. One out of four respondents reported an infusion time of 65 minutes or less, possibly due to shifting to nivolumab only maintenance phase of the nivolumab+ipilimumab regimen and/or using a shorter infusion period for the ipilimumab component (consistent with FDA labeling as a single agent).

We compared the reported average patient wait time to other estimates from the literature. The wait time reported by patients in our study (57 minutes on average) aligns with results from a Texas ambulatory treatment center, reporting 53.3 minutes of wait in 2010 after an intervention to reduce wait was implemented (from 72.7 minutes before the intervention) [2]. Our results also align with those of a recent survey from the NCCN Infusion Efficiency Workgroup, which reported an average waiting time of 58 minutes per patient receiving chemotherapy [1]. These different studies indicate that currently in the U.S., patients typically wait at the infusion center for about an hour before undergoing their cancer treatment.

Most patients reported having a caregiver (61 [61.0%]), either paid (41 [41.0%]) or unpaid (20 [20.0%]), suggesting that the infusion-related time lost may extend to family members and friends. Patients supported by paid caregivers needed an average of 5.6 hours of paid care per infusion visit over 3 days. This cost is in addition to the expenses relating to public or private transportation, overnight stay accommodation and co-payments paid for the visit itself, emphasizing how infusion visits result in a considerable time commitment and financial burden for patients.

The substantial amount of time required from oncologists and nurses for each infusion visit highlights the potential for optimizing the use of oncology professionals and facilities by shifting towards an immunotherapy regimen requiring less frequent dosing. While efficiency improvements may be achieved through multiple routes (e.g., improved patient scheduling or flexible allocation of resources within the center) [4], using treatment regimens that require less frequent administration can improve the efficient use of resources without the need for major changes. Such a shift could also improve patients’ satisfaction by reducing the amount of time they and their caregivers would spend on extra infusions and recovery time, and may result in monetary savings as well. Less frequent immunotherapy infusions also decrease the frequency with which patients travel to infusion centers and reduce their exposure time at the center, thereby lowering their risks of acquiring infections [16], of particular relevance during the COVID-19 pandemic. As suggested by responses from health care professionals on the impact of COVID-19 on immunotherapy infusions, close management of infectious disease transmission is burdensome in the context of oncology care delivery and may result in a reduction in the number of patients undergoing or returning for immunotherapy.

The results of our study suggest that it may be possible to reduce total infusion time per treatment course as the duration of time is similar per infusions, but the number of infusions are reduced when pembrolizumab Q6W is administered. This was further investigated in a separate study that utilized the results of this study with the expected number of infusion visits both by a patient over their treatment course and within an infusion center over 1 year to identify the potential magnitude of savings in time and productivity [17]. This separate study demonstrated that Q6W could substantially reduce the number of infusion visits and associated chair time required over the duration of a treatment course, reducing the time and monetary burden for patients and their caregivers; thus, with fewer infusions over their treatment course, savings in time should be realized. Therefore, shifting to a less frequent dosing regimen such as pembrolizumab Q6W has potential to reduce and save time spent by nurses, oncologists and patients during an infusion visit.

### Limitations

Our study has several limitations. First, although numerical results suggest no meaningful difference in time per infusion visit with either immunotherapy, the study design (the small sample size and no dosing-specific questions for physicians) limited the power of our study. Since survey questions posed to oncologists did not differ based on treatment dosing frequency, it is possible that differences in physician’s consultation time for patients with different dosing frequencies existed but were not captured. However, patients did not report a longer infusion visit overall (and for the infusion itself). Moreover, nurses are reporting similar durations. We found no major time differences per infusion event based on the dosing frequency of each regimen. Our study was not designed to look at factors considered when choosing one dosing schedule over the other, though this could be an area of future research.

Second, the surveys only focused on infusions related to the administration of pembrolizumab, nivolumab, or nivolumab+ipilimumab, excluding other treatment options. However, these are currently the most widely utilized immunotherapies for the treatment of melanoma in the U.S. [18] Third, results from the patient survey may not be representative of the typical patient with melanoma undergoing immunotherapy treatment in US. The survey relied on online panel recruitment and thus is based on non-probability sampling. Our sample may not be representative of the general melanoma patient population, as the respondents were 41.8 years-old on average, and 87% were males. Thus, patients in the study were younger (41.8 vs 65 years of age) and more likely to be male (87% vs 60%) than the typical patient with melanoma in the U.S. (65 years-old on average and 60% being males) [19]. Furthermore, all respondents were employed. Consequently, our results regarding work loss can only be interpreted for patients within the workforce. Fourth, any retrospective survey is subject to recall bias; participants may not remember previous events accurately. Thus, results likely reflect respondents’ perceptions of time rather than actual times. Respondents might also have misinterpreted the questions, not reporting the outcome measures that were expected; such may be the case for those responses that were excluded for implausible time values. However, to reduce the likelihood of misinterpretation, a pilot of the questionnaires was conducted and improvements in the wording of each survey were made. Additionally, nurses may have provided time estimates during which they attend more than one patient at a time, and oncologists may have provided time spent by patients or nurses rather than only the time they directly spend with a patient. Estimating these times using electronic medical records could provide a more accurate estimate of the actual time spent by oncologists, nurses and patients per infusion. Nevertheless, after unusual or extreme responses were discarded, estimated times were mostly consistent with the recommended dosages [7, 9] and what is expected to be observed in U.S. clinical practice [1]. Finally, collecting adverse event information was out of the scope of the survey, so any potential differences in the need for additional visits to the doctors were not captured here.

## Conclusion

Our study indicates that each immunotherapy infusion visit requires several hours of work from oncologists and nurses and represents a substantial burden for patients with melanoma and their caregivers. The use of immunotherapies with less frequent dosing, such as pembrolizumab Q6W, can improve the efficient use of resources by infusion centers and health care professionals, and reduce the time and productivity loss burden for patients.

## Supplementary Information

**Additional file 1. ** Patient survey. Physician Survey. Nurse Survey.

**Additional file 2: Figure S1.** Mean, minimum, and maximum time oncology nurses spend per patient and infusion visit by different dosing schedules. *P*-values assess if differences by immunotherapy and dose are significant. **Figure S2.** Average, minimum, and maximum time patients with melanoma spend per infusion visit by different dosing schedules for each phase of the infusion visit.

## Data Availability

The datasets used and/or analyzed during the current study are available from the corresponding author on reasonable request.
