# Supplementary material for: Time and productivity loss associated with immunotherapy infusions for the treatment of melanoma in the United States: a survey of health care professionals and patients
Source: BMC Health Serv Res. 2023 Feb 9;23:136. doi: 10.1186/s12913-022-08904-4 (PMC9910242; doi:10.1186/s12913-022-08904-4)
Supplement: Supplementary file 1 — Additional file 1. Patient survey. Physician Survey. Nurse Survey. [file 12913_2022_8904_MOESM1_ESM.pdf]

Table of Contents

Patient survey 2
Physician Survey 11
Nurse Survey 17

Patient survey

1. Do you agree to take part in this survey?
   a. Yes
   a. No **[TERMINATE]**

2. Do you currently have or have been diagnosed with melanoma?
   a. Yes
   b. No **[TERMINATE]**

3. Do you currently have or have been diagnosed with any other cancer apart from melanoma?
   a. Yes [**TERMINATE]**
   b. No

4. Are you receiving your treatment after having surgery (complete removal of cancer tumor) for your melanoma?
   a. Yes
   b. No
   [SKIP QUESTION 5 IF ANSWER IS "Yes"]

5. Are you receiving your treatment because your disease came back after being treated previously (metastatic)?
   a. Yes
   b. No
   [IF RESPONDENT ANSWERS 'NO' TO QUESTION 4 AND QUESTION 5, THEN TERMINATE SURVEY]

6. Are you an adult?
   a. Yes
   b. No **[TERMINATE]**

7. What is the current stage of your melanoma?
   a. Stage I or Stage II **[TERMINATE]**
   b. Stage III
   c. Stage IV
   d. I do not know **[TERMINATE]**

8. Are you currently receiving any of the following treatments for your melanoma?
   a. Keytruda^{TM} (Pembrolizumab)
   b. Only Opdivo^{TM} (Nivolumab)
   c. Combination therapy: Opdivo^{TM}+ Yervoy^{TM} (Nivolumab + Ipilimumab)
   d. None of the above **[TERMINATE]**
   e. I do not know **[TERMINATE]**
   f. Other, please specify__________**[TERMINATE]**

9. How frequently, on an average, do you go to the clinic for your infusion treatment?
   a. Once every 2 weeks
   b. Once every 3 weeks
   c. Once every 4 weeks
   d. Once every 6 weeks
   e. Other, please specify ___________

10. What is the name of the infusion center you visit?

    ____

11. What is your gender?
    a. Male
    b. Female
    c. Other
    d. Prefer not to answer

12. What state are you currently residing in?

____

13. What is your age?

    _____________ Years

The questions in the next section require generalization of time estimates. Please provide average time spent or time spent in most cases by you during your infusion visits.

14. Are you currently working for pay at a job or business?

    a. Yes

    b. No

    [SKIP QUESTION 15, 16, 17 ABOUT WORKING HOURS IF ANSWER IS “No"]

15. How much paid work time do you usually miss ON THE DAY BEFORE your treatment/infusion? If you do not miss any time on paid work, please enter “0” in both cells to indicate 0 hours 0 minutes. Please enter “0” for hours if less than 60 minutes.

    ____ hours ____ minutes

16. How much paid work time do you usually miss ON THE DAY OF your treatment/infusion? If you do not miss any time on paid work, please enter “0” in both cells to indicate 0 hours 0 minutes. Please enter “0” for hours if less than 60 minutes.

    ____ hours ____ minutes

17. How much paid work time do you usually miss ON THE DAY AFTER your treatment/infusion? If you do not miss any time on paid work, please enter “0” in both cells to indicate 0 hours 0 minutes. Please enter “0” for hours if less than 60 minutes.

    ____ hours ____ minutes

18. How much time do you usually miss on household chores or unpaid work ON THE DAY BEFORE your treatment/infusion? If you do not miss any time on household chores or

unpaid work, please enter “0” in both cells to indicate 0 hours 0 minutes. Please enter “0” for hours if less than 60 minutes.

____ hours ____ minutes

19. How much time do you usually miss on household chores or unpaid work ON THE DAY OF your treatment/infusion? If you do not miss any time on household chores or unpaid work, please enter “0” in both cells to indicate 0 hours 0 minutes. Please enter “0” for hours if less than 60 minutes.

    ____ hours ____ minutes

20. How much time do you usually miss on household chores or unpaid work ON THE DAY AFTER your treatment/infusion? If you do not miss any time on household chores or unpaid work, please enter “0” in both cells to indicate 0 hours 0 minutes. Please enter “0” for hours if less than 60 minutes.

    ____ hours ____ minutes

21. How much time do you usually miss on leisure activities ON THE DAY BEFORE your treatment/infusion? If you do not miss any time on leisure activities, please enter “0” in both cells to indicate 0 hours 0 minutes. Please enter “0” for hours if less than 60 minutes?

    ____ hours ____ minutes

22. How much time do you usually miss on leisure activities ON THE DAY OF your treatment/infusion? If you do not miss any time on leisure activities, please enter “0” in both cells to indicate 0 hours 0 minutes. Please enter “0” for hours if less than 60 minutes?

    ____ hours ____ minutes

23. How much time do you usually miss on leisure activities ON THE DAY AFTER your treatment/infusion? If you do not miss any time on leisure activities, please enter “0” in both cells to indicate 0 hours 0 minutes. Please enter “0” for hours if less than 60 minutes?

    ____ hours ____ minutes

24. How far is the infusion center from your home?

________________miles

25. What is the total travel time that you spend going **to and from** the infusion center?

______hours ________ minutes

26. What is the mode of transport you typically use to travel to and from the infusion center?

a. Private Transport (self, friend, or caregiver)

b. Public Transport (bus, train, etc.)

c. Cab/Taxi/Uber/Lyft/car sharing Service

d. Other, please specify __________

27. Have you stayed overnight near the infusion center in order to get your treatment?

a. Never

b. Always

c. Sometimes, please specify the % of infusions with overnight stay

___________________%

[SKIP QUESTION 28 IF ANSWER IS "Never"]

28. If you need to stay overnight, what type of accommodation do you generally use?

a. Hotel/Motel/short term rental

b. Family/Friend

c. Provided by hospital

d. Other, please specify_________

The next set of questions is regarding the time spent at the infusion center during different phases. If you do not spend any time for a particular phase, please enter "0" in both boxes to indicate 0 hours 0 minutes. Please enter "0" for hours if less than 60 minutes.

29. What is the total time you spend at the infusion center (from when you step into the infusion center to when you step out)?

______hours ________ minutes

30. How long is the average wait time *before* your appointment starts (from when you step into the infusion center to when you are seen by a nurse or physician)?

______hours ________ minutes

31. What is the average time you spend from when the appointment starts to when you sit in an infusion chair?

______hours ________ minutes

32. What is the average time you spend in the infusion chair?

______hours______ minutes

33. How much additional time on average do you spend at the infusion center once the infusion is complete?

______hours ________ minutes

34. Do you have to undergo a blood test before infusion?
    a. Never
    b. Always
    c. Sometimes, please specify the % of times you undergo a blood test__________%

35. Do you receive care from a caregiver on the day of OR on the day before/after the infusion to complete the tasks which you missed due to your infusion visit?
    a. Yes
    b. No

    [SKIP QUESTIONS 36-42 IF ANSWER IS "No"]

36. What type of caregiver do you use?
    a. Unpaid caregiver/Family/Friend

b. Paid caregiver
c. Both. Please specify the % of times you use unpaid caregiver_________%

[SKIP QUESTIONS 40-42 IF ANSWER IS “a. Unpaid Caregiver”; SKIP QUESTIONS 37-39 IF ANSWER IS “b. Paid Caregiver”]

37. How much time does your unpaid caregiver miss ON THE DAY of the infusion on the following activities because of the infusion?
    a. Leisure time __________________ hours _______minutes
    b. Paid Work time __________________ hours _______minutes
    c. Household chores or unpaid work ___________________hours _______minutes

38. How much time does your unpaid caregiver miss on the DAY BEFORE the infusion on the following activities because of the infusion?
    a. Leisure time __________________ hours _______minutes
    b. Paid Work time __________________ hours _______minutes
    c. Household chores or unpaid work ___________________hours _______minutes

39. How much time does your unpaid caregiver miss on the DAY AFTER the infusion on the following activities because of the infusion ?
    a. Leisure time __________________ hours _______minutes
    b. Paid Work time __________________ hours _______minutes
    c. Household chores or unpaid work ___________________hours _______minutes

40. How much time does your paid caregiver spend with you due to the infusion ON THE DAY of the infusion?
    ___________________hours _______minutes

41. How much time does your paid caregiver spend with you due to the infusion on the DAY BEFORE the infusion?
    ___________________hours _______minutes

42. How much time does your paid caregiver spend with you due to the infusion on the DAY AFTER the infusion?

___________________hours _______minutes

43. Are you facing any difficulties while booking an appointment for the infusion due to COVID?

   a. Yes. Please specify______
   b. No

44. Has the mode of travel and travel time to infusion center changed due to COVID?

a. Yes, how? ___________

Please specify how much more/less time it does take you now to travel to and from the infusion center

	Hours	Minutes
Increase		
Decrease		

b. No

45. Has the waiting time at the infusion center, before being attended to by a nurse or physician, changed due to COVID?

   a. Yes, Please specify how much more/less time now do you have to wait at an infusion center

	Hours	Minutes
Increase		
Decrease		

   b. No

46. Has the availability of a caregiver been impacted due to COVID?

   a. Yes (If yes, how? ____________)
   b. No

This ends the survey.

Thank you again for your assistance!

Physician Survey

1. Do you agree to participate in this survey?
   a. Yes
   b. No **[TERMINATE]**

2. What role do you perform at the infusion center?
   a. Physician/ Oncologist
   b. None of the above **[TERMINATE]**

3. Please check below the types of patients you are currently treating or attending (check all that apply):
   a. Melanoma patients receiving adjuvant therapy
   b. Melanoma patients receiving therapy for advanced or metastatic disease
   c. None of the above **[TERMINATE]**

4. Are you currently administering any of the following therapies for your melanoma patients? (**Check *all that apply)***
   a. Keytruda™ (Pembrolizumab)
   b. Opdivo™ (Nivolumab)
   c. Combination therapy: Opdivo+ Yervoy™ (Nivolumab + Ipilimumab)
   d. All of the above
   e. None of the above **[TERMINATE]**
   [ONLY THOSE THERAPIES SELECTED WILL BE INCLUDED AS OPTIONS IN SUBSEQUENT QUESTIONS]

The next question is about the treatment criteria.

5. What factors do you take into consideration when selecting an immunotherapy regimen for your melanoma patients?
   ____

The next set of questions is about the consultation and time for a typical infusion episode by an oncologist.

6. How often are patients required to undergo a blood test before starting their infusion?
   a. Never
   b. For first infusion only
   c. Always
   d. Sometimes. Please specify the % of times a blood test is required before starting infusion_______

7. How much time, on average, does it take for **you** to provide consultation to patient undergoing infusion for the **first time** on the following therapies:
   a. Keytruda^{TM} (Pembrolizumab) ________hours _________minutes
   b. Opdivo^{TM} (Nivolumab) ________hours _________minutes
   c. Combination therapy: Opdivo+ Yervoy^{TM} (Nivolumab + Ipilimumab) ________hours _________minutes

8. Do you consult with **returning** patients at/before **every** infusion visit?
   a. Yes
   b. No, please specify % of times __________%

9. How much time, on average, does it take for **you** to provide consultation to a ***returning*** immunotherapy infusion patient on the following (Please enter "0" for hours if less than 60 minutes):
   a. Keytruda^{TM} (Pembrolizumab) ________hours _________minutes
   b. Opdivo^{TM} (Nivolumab) ________hours _________minutes
   c. Combination therapy: Opdivo+ Yervoy^{TM} (Nivolumab + Ipilimumab) ________hours _________minutes

10. How much time, on average, do ***you*** spend with a patient during their ***premed/hydration*** for the following (If you do not spend any time with the patient during this phase, please

enter “0” in both blanks to indicate 0 hours 0 minutes. Please enter “0” for hours if less than 60 minutes):

a. Keytruda™ (Pembrolizumab) ________hours _________minutes

b. Opdivo™ (Nivolumab) ________hours _________minutes

c. Combination therapy: Opdivo+ Yervoy™ (Nivolumab + Ipilimumab) ________hours _________minutes

11. How much time, on average, do **you** spend with a patient **during** their **infusion** for the following (If you do not spend any time with the patient during this phase, please enter “0” in both blanks to indicate 0 hours 0 minutes. Please enter “0” for hours if less than 60 minutes):

    a. Keytruda™ (Pembrolizumab) ________hours _________minutes

    b. Opdivo™ (Nivolumab) ________hours _________minutes

    c. Combination therapy: Opdivo+ Yervoy™ (Nivolumab + Ipilimumab) ________hours _________minutes

12. How much time, on average, do ***you*** spend with a patient during their ***post-infusion observation*** for the following (If you do not spend any time with the patient during this phase, please enter “0” in both blanks to indicate 0 hours 0 minutes. Please enter “0” for hours if less than 60 minutes):

    a. Keytruda™ (Pembrolizumab) ________hours _________minutes

    b. Opdivo™ (Nivolumab) ________hours _________minutes

    c. Combination therapy: Opdivo+ Yervoy™ (Nivolumab + Ipilimumab) ________hours _________minutes

13. How much time, on average, do ***you*** spend with a patient during their ***post-infusion hydration*** for the following (If you do not spend any time with the patient during this phase, please enter “0” in both blanks to indicate 0 hours 0 minutes. Please enter “0” for hours if less than 60 minutes)

    a. Keytruda™ (Pembrolizumab) ________hours _________minutes

    b. Opdivo™ (Nivolumab) ________hours _________minutes

c. Combination therapy: Opdivo+ Yervoy™ (Nivolumab + Ipilimumab) ________hours _________minutes

14. Do you spend time on any other activities not specified earlier related to the patient's infusion visit?

    a. Yes, please specify which activities_____

    b. No

    [SKIP QUESTION 15 IF ANSWER IS "No"]

15. Please specify below the time you spend on the additional **activities specified in the previous question**, for the following treatments during their infusion visit (Please enter "0" for hours if less than 60 minutes):

    a. Keytruda™ (Pembrolizumab) ________hours _________minutes

    b. Opdivo™ (Nivolumab) ________hours _________minutes

    c. Combination therapy: Opdivo+ Yervoy™ (Nivolumab + Ipilimumab) ________hours _________minutes

The next set of questions is about the infusion center.

16. What type of facility do you primarily treat your melanoma patients at?

    a. Academic medical center (teaching hospital)

    b. Private hospital

    c. Community hospital

    d. Private practice

    e. Other, please specify ___________

17. In which state is your facility located?

    ____

18. Does your infusion center participate in the Oncology Care Model?

    a. Yes

    b. No

c. I do not know

19. How many chairs are available at the infusion center?

a. ____________________

b. I do not know

The next set of questions is about the impact of COVID.

20. Has the onset of the COVID-19 pandemic impacted treatment of infusion patients in any way?

a. Yes.

a. No

[SKIP REMAINING QUESTIONS IF ANSWER IS "No"]

21. Has there been a change in your time requirements associated with any of the steps in the infusion process (described previously) as a consequence of COVID-19?

a. Yes

b. No

[SKIP QUESTION 22 IF ANSWER IS "No"]

22. Please specify how your time associated with each of the steps below in the table has changed (Please enter "0" in both columns if no change. If there is a change, enter value in the cell as applicable, and put "0" in the opposite cell):

Activity	Increase (minutes)	Decrease (minutes)
Consultation time		
Premed/hydration time		
Infusion time		
Post infusion observation time		
Post infusion hydration time		
Any other activity		

23. Has the number of slots available for appointment for infusion patients changed as a consequence of COVID-19?
    a. Yes, please specify ______________ % increase ______________ % decrease
    b. No

24. Has the number of **new** patients undergoing immunotherapy infusion changed as a consequence of COVID-19?
    a. Yes, please specify______________ % increase ______________ % decrease
    b. No

25. Has the number of **returning** patients undergoing immunotherapy infusion changed as a consequence of COVID-19?
    a. Yes, please specify______________ % increase ______________ % decrease
    b. No

26. Is there any other way COVID has impacted a typical infusion episode?
    ____

This ends the survey.
Thank you again for your assistance!

Nurse Survey

1. Do you agree to participate in this survey?
   a. Yes
   b. No **[TERMINATE]**

2. What role do you perform at the infusion center?
   a. Nurse or Nurse Manager
   b. None of the above **[TERMINATE]**

3. Please check below the types of patients you are currently treating or attending (check all that apply):
   a. Melanoma patients receiving adjuvant therapy
   b. Melanoma patients receiving therapy for advanced or metastatic disease
   c. None of the above **[TERMINATE]**

4. Are you currently administering any of the following therapies for your melanoma patients? (**Check *all that apply)***
   a. Keytruda^{TM} (Pembrolizumab)
   b. Opdivo^{TM} (Nivolumab)
   c. Combination therapy: Opdivo+ Yervoy^{TM} (Nivolumab + Ipilimumab)
   d. All of the above
   e. None of the above **[TERMINATE]**
   [ONLY THOSE THERAPIES SELECTED WILL BE INCLUDED AS OPTIONS IN SUBSEQUENT QUESTIONS]

The next set of questions is about average time involved in administering/monitoring for a typical infusion episode by a nurse/nurse manager.

5. On average, how long does it take for you to check the patient in and do vitals? Please enter “0” for hours if less than 60 minutes.
   a. ______hours and ________ minutes

b. Not Applicable

6. How often are patients required to undergo a blood test before starting their infusion?
   a. Always
   b. Never
   c. Sometimes. Please specify the % of times a blood test is required before starting an infusion _______

7. Do you draw blood for the blood test?
   a. Yes
   b. No
   [SKIP QUESTION 8 IF ANSWER IS "No"]

8. How much time does it take you to draw blood for the test? Please enter "0" for hours if less than 60 minutes.
   ______hours ________ minutes

9. How much time, on average, do **you spend preparing a patient prior to the infusion** with the following: (If you do not spend any time with the patient during this phase, please enter "0" in both blanks to indicate 0 hours 0 minutes. Please enter "0" for hours if less than 60 minutes).
   a. Keytruda^{TM} (Pembrolizumab) 200 mg ________hours _________minutes
   b Keytruda^{TM} (Pembrolizumab) 400 mg ________hours _________minutes
   c. Opdivo^{TM} (Nivolumab) 240 mg _______hours _________minutes
   d. Opdivo^{TM} (Nivolumab) 480 mg ________hours _________minutes
   e. Combination therapy: Opdivo+ Yervoy^{TM} (Nivolumab + Ipilimumab): Nivolumab 1 mg/kg followed by Ipilimumab 3 mg/kg ________hours _________minutes

10. Once the infusion starts, how much time, on average, do **you spend** with a patient **during** their **premed/hydration** for the following (If you do not spend any time with the patient during this phase, please enter "0" in both blanks to indicate 0 hours 0 minutes. Please enter "0" for hours if less than 60 minutes):

a. Keytruda™ (Pembrolizumab) 200 mg ________hours _________minutes

b. Keytruda™ (Pembrolizumab) 400 mg ________hours _________minutes

c. Opdivo™ (Nivolumab) 240 mg _______hours _________minutes

d. Opdivo™ (Nivolumab) 480 mg ________hours _________minutes

e. Combination therapy: Opdivo+ Yervoy™ (Nivolumab + Ipilimumab): Nivolumab 1 mg/kg followed by Ipilimumab 3 mg/kg ________hours _________minutes

11. How much time, on average, do **you spend** with a patient **during** their **immunotherapy infusion** for the following (If you do not spend any time with the patient during this phase, please enter "0" in both blanks to indicate 0 hours 0 minutes. Please enter "0" for hours if less than 60 minutes):

    a. Keytruda™ (Pembrolizumab) 200 mg ________hours _________minutes

    b. Keytruda™ (Pembrolizumab) 400 mg ________hours _________minutes

    c. Opdivo™ (Nivolumab) 240 mg _______hours _________minutes

    d. Opdivo™ (Nivolumab) 480 mg ________hours _________minutes

    e. Combination therapy: Opdivo+ Yervoy™ (Nivolumab + Ipilimumab): Nivolumab 1 mg/kg followed by Ipilimumab 3 mg/kg ________hours _________minutes

12. How much time, on average, do **you spend** with a patient during their **post immunotherapy hydration** for the following (If you do not spend any time with the patient during this phase, please enter "0" in both blanks to indicate 0 hours 0 minutes. Please enter "0" for hours if less than 60 minutes):

    a. Keytruda™ (Pembrolizumab) 200 mg ________hours _________minutes

    b. Keytruda™ (Pembrolizumab) 400 mg ________hours _________minutes

    c. Opdivo™ (Nivolumab) 240 mg _______hours _________minutes

    d. Opdivo™ (Nivolumab) 480 mg ________hours _________minutes

    e. Combination therapy: Opdivo+ Yervoy™ (Nivolumab + Ipilimumab): Nivolumab 1 mg/kg followed by Ipilimumab 3 mg/kg ________hours _________minutes

13. How much time, on average, do **you spend** on **patient observation after completing their entire infusion** with the following during **the first cycle** (If you do not spend any time

with the patient during this phase, please enter “0” in both blanks to indicate 0 hours 0 minutes. Please enter “0” for hours if less than 60 minutes.):

a. Keytruda™ (Pembrolizumab) 200 mg ________hours _________minutes

b. Keytruda™ (Pembrolizumab) 400 mg ________hours _________minutes

c. Opdivo™ (Nivolumab) 240 mg _______hours _________minutes

d. Opdivo™ (Nivolumab) 480 mg ________hours _________minutes

e. Combination therapy: Opdivo+ Yervoy™ (Nivolumab + Ipilimumab): Nivolumab 1 mg/kg followed by Ipilimumab 3 mg/kg ________hours _________minutes

14. How much time, on average, do **you spend** on **patient observation after completing their entire infusion** with the following for **subsequent cycles** (If you do not spend any time with the patient during this phase, please enter “0” in both blanks to indicate 0 hours 0 minutes. Please enter “0” for hours if less than 60 minutes.):

a. Keytruda™ (Pembrolizumab) 200 mg ________hours _________minutes

b. Keytruda™ (Pembrolizumab) 400 mg ________hours _________minutes

c. Opdivo™ (Nivolumab) 240 mg _______hours _________minutes

d. Opdivo™ (Nivolumab) 480 mg ________hours _________minutes

e. Combination therapy: Opdivo+ Yervoy™ (Nivolumab + Ipilimumab): Nivolumab 1 mg/kg followed by Ipilimumab 3 mg/kg ________hours _________minutes

15. Do you spend time on any other activities not specified earlier?

a. Yes, please specify the activities_____

b. No

[SKIP QUESTION 16 IF ANSWER IS “No”]

16. Please specify how much **time you spend on these activities** with/for the patient receiving the following, during their infusion visit (Please enter “0” for hours if less than 60 minutes):

a. Keytruda™ (Pembrolizumab) 200 mg ________hours _________minutes

b. Keytruda™ (Pembrolizumab) 400 mg ________hours _________minutes

c. Opdivo™ (Nivolumab) 240 mg _______hours _________minutes

d. Opdivo™ (Nivolumab) 480 mg ________hours _________minutes

e. Combination therapy: Opdivo+ Yervoy™ (Nivolumab + Ipilimumab): Nivolumab 1 mg/kg followed by Ipilimumab 3 mg/kg ________hours _________minutes

The next set of questions is about the infusion center.

17. What type of facility do you primarily treat your melanoma patients at?
    a. Academic medical center (teaching hospital)
    b. Private hospital
    c. Community hospital
    d. Private practice
    e. Other, please specify ___________

18. In which state is your facility located?

    ____

19. Does your infusion center participate in the Oncology Care Model?
    a. Yes
    b. No
    c. I do not know

20. How many chairs are available at the infusion center?

    ____

    b. I do not know

21. How many hours does the infusion center operate per day?
    _______________hours

22. How many of the total daily operating hours are peak hours?

    _______________hours

23. How would you define/describe 'peak hours' for the infusion center?

    ____

24. What % of the chairs, on average, are filled during peak hours?

    a.________________%

    b. I Don't Know

25. What % of the chairs, on average, are filled during off-peak hours?

    a. _________________%

    b. I Don't Know

26. On average, how many patients do you see in a day?

    ____

27. Out of the total patients, how many of these are seen during peak hours?

    ____

The next set of questions is about the impact of COVID.

28. Has the onset of the COVID-19 pandemic impacted treatment of infusion patients in any way?

    a. Yes.

    a. No

    [SKIP REMAINING QUESTIONS IF ANSWER IS "No"]

29. Has there been a change in your time requirements associated with any of the steps in the infusion process (described previously) as a consequence of COVID-19?

    a. Yes

    b. No

    [SKIP QUESTION 30 IF ANSWER IS "No"]

30. Please specify how your time associated with each of the steps below in the table has changed (Please enter “0” in both columns if no change. If there is a change, enter value in the cell as applicable, and put “0” in the opposite cell):

Activity	Increase (minutes)	Decrease (minutes)
Consultation time		
Premed/hydration time		
Infusion time		
Post infusion observation time		
Post infusion hydration time		
Any other activity		

31. Has the number of slots available for appointment for infusion patients changed as a consequence of COVID-19?
    a. Yes, please specify ______________ % increase ______________ % decrease
    b. No

32. Has the number of **new** patients undergoing immunotherapy infusion changed as a consequence of COVID-19?
    a. Yes, please specify______________ % increase ______________ % decrease
    b. No

33. Has the number of **returning** patients undergoing immunotherapy infusion changed as a consequence of COVID-19?
    a. Yes, please specify______________ % increase ______________ % decrease
    b. No

34. What % of chairs were filled prior to COVID-19?
    a. _________________% during peak hours
    b. _________________% during off-peak hours

35. Is there any other way COVID has impacted a typical infusion episode?

____

This ends the survey.
Thank you again for your assistance!
