# Supplementary material for: Time and productivity loss associated with immunotherapy infusions for the treatment of melanoma in the United States: a survey of health care professionals and patients
Source: BMC Health Serv Res. 2023 Feb 9;23:136. doi: 10.1186/s12913-022-08904-4 (PMC9910242; doi:10.1186/s12913-022-08904-4)

**Supplementary File 2**

Figure S1. Mean, minimum, and maximum time oncology nurses spend per patient and infusion visit by different dosing schedules. P-values assess if differences by immunotherapy and dose are significant.


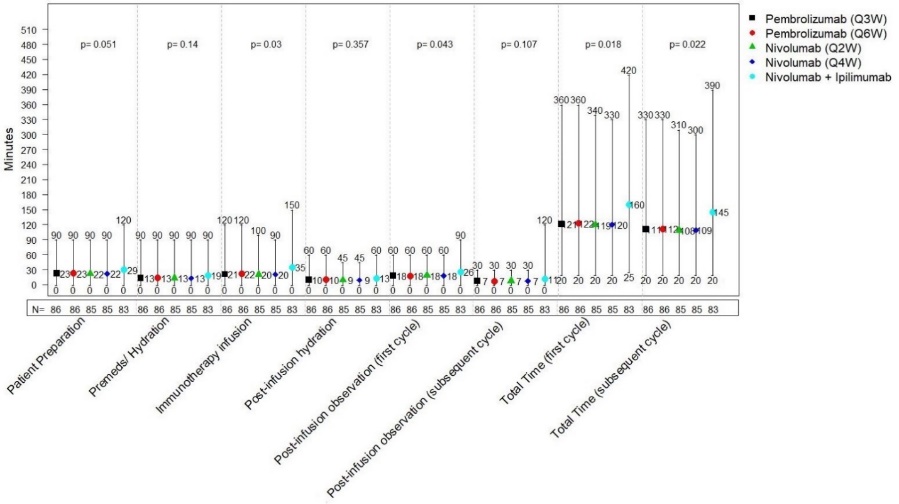


Figure S2. Average, minimum, and maximum time patients with melanoma spend per infusion visit by different dosing schedules for each phase of the infusion visit


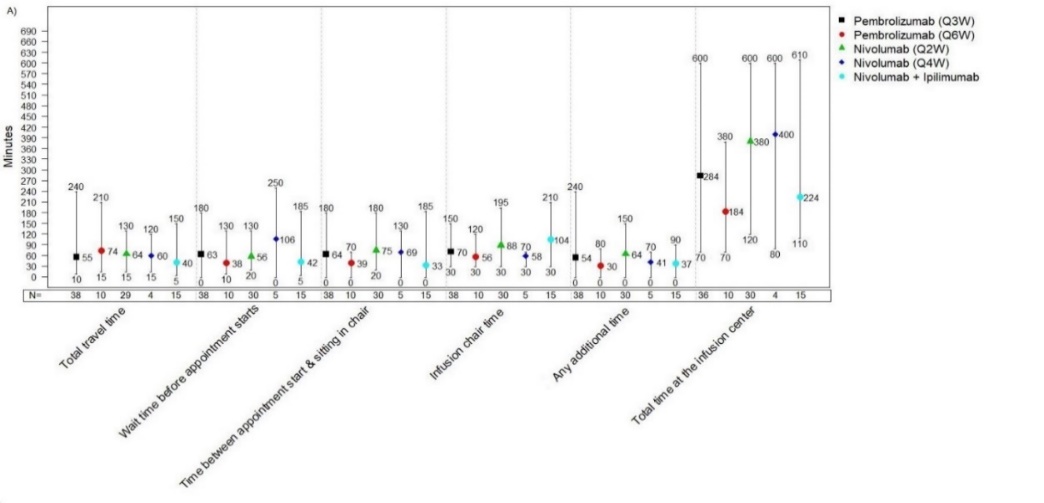

Supplement: Supplementary file 2 — Additional file 2: Figure S1. Mean, minimum, and maximum time oncology nurses spend per patient and infusion visit by different dosing schedules. P-values assess if differences by immunotherapy and dose are significant. Figure S2. Average, minimum, and maximum time patients with melanoma spend per infusion visit by different dosing schedules for each phase of the infusion visit. [file 12913_2022_8904_MOESM2_ESM.docx]
